# Facile Photochemical Syntheses of Conjoined Nanotwin Gold-Silver Particles within a Biologically-Benign Chitosan Polymer

**DOI:** 10.3390/nano9040596

**Published:** 2019-04-11

**Authors:** Daniel K. Korir, Bharat Gwalani, Abel Joseph, Brian Kamras, Ravi K. Arvapally, Mohammad A. Omary, Sreekar B. Marpu

**Affiliations:** 1 Department of Chemistry, University of North Texas, Denton, TX 76203, USA; DanielKorir@my.unt.edu (D.K.K.); AbelJoseph2@my.unt.edu (A.J.); BrianKamras@my.unt.edu (B.K.); RaviArvapally@my.unt.edu (R.K.A.); 2 Department of Materials Science and Engineering, University of North Texas, Denton, TX 76203, USA; BharatGwalani@my.unt.edu

**Keywords:** isotropic, anisotropic, photochemical, bifunctional, nanomaterials, plasmonic

## Abstract

A simple photochemical method for making conjoined bi-metallic gold-silver (Au/Ag) nanotwins, a new breed of nanoparticles (NPs), is developed. To the best of our knowledge, the photochemical method resulted in distinct, conjoined, bimetallic nanotwins that are different from any well-established alloyed or core-shell nanostructures in the literature. The conjoined Au-Ag NPs possessed surface plasmon resonance (SPR) properties of both metals. The bimetallic nanostructures possessing distinctive optical properties of both metals were obtained using Au NPs as seeds in the first step, followed by the addition of a silver precursor as feed in the second step during a photochemical irradiation process. In the first step, small, isotropic or large, anisotropic Au NPs are generated by photoinduced reduction within a biocompatible chitosan (CS) polymer. In the second step, a silver precursor (AgNO_3_) is added as the feed to the AuNPs seed, followed by irradiation of the solution in the ice-bath. The entire photochemical irradiation process resulting in the formation of bimetallic Au-AgNPs did not involve any other reducing agents or stabilizing agents other than the CS polymer stabilizer. The small, conjoined Au-Ag bi-metallic NPs exhibited SPR with peak maxima centering at ~400 nm and ~550 nm, whereas the large conjoined nanoparticles exhibited SPR with peak maxima centering at ~400 nm, 550 nm, and 680 nm, characteristic of both gold and silver surface plasmons in solution. The tunability in the SPR and size of the bimetallic NPs were obtained by varying the reaction time and other reaction parameters, resulting in average sizes between 30 and 100 nm. The SPR, size, distribution, and elemental composition of the bi-metallic NPs were characterized using UV-Vis absorption, electron microscopy, and energy dispersive X-ray spectroscopy (EDS) studies.

## 1. Introduction

In this report, we report a facile photochemical process that results in the formation of hybrid gold-silver (Au-Ag) nanoparticles (NPs) following a two-step photoreduction protocol. Metal particles in the nanoscale dimension are known to exhibit distinctive optical properties, which can be tuned by changing their shape, size, surface charge, and composition [1]. Ag- and Au-based metallic NPs, along with their bimetallic alloyed and core-shell structures, are widely-studied nanomaterials [2,3,4] whose plasmonic and physicochemical properties are exploited for applications such as sensors, antibacterial agents, cancer therapy, and catalysis [5,6]. Selection of surface chemistry, reducing agents, stabilizing agents, and functionalization of nanoparticles is dictated by the application of the NPs. Nanomaterials designed for biomedical applications, for example, have to be synthesized without toxic reagents among reducing agents and/or stabilizers (e.g., sodium borohydride and cetyltrimethylammonium bromide (CTAB), respectively) or they have to be processed to effectively remove such reagents prior to use. Green chemistry techniques embracing environmentally-friendly synthetic routes for producing inorganic and organic NPs using natural sources such as plant and animal extracts or utilizing microorganisms, for example algae, fungi, and yeast, to form NPs are well established [7]. On the other hand, NPs destined for catalytic applications are designed to be interfaced with metal-oxide supports irrespective of the presence of the abovementioned reducing agents or stabilizers. Compared to monometallic systems, alloyed bimetallic NPs or core-shell structures are known to have enhanced catalytic activity even at low temperatures and concentrations [7]. Superior catalytic performance of bimetallic NPs is attributed to electronic coupling between the two metal NPs [8].

Among recent investigations on bioengineered nanomaterials for applications in cancer therapies, silver and gold nanoparticles are specifically designed for diagnosis, imaging, and photothermal therapy (PTT) via hyperthermia of cancer cells for both in vivo and in vitro models [9]. In addition, AgNPs have been reported to be cytotoxic to cancerous cells at concentrations not cytotoxic to healthy cells. Therefore, combining AgNPs and AuNPs in a single therapy to exploit both the cytotoxicity of AgNPs and heat generation of AuNPs is highly relevant for anticancer therapy applications [10]. Additionally, in light of the multidrug resistance (MDR) of most cancer cells, multifunctional nanoparticles could provide more versatile therapeutic functions given the lack of need for chemotherapy drugs upon invoking PTT. Further, cancer cells are known to overexpress heat shock proteins, rendering PTT alone inefficient [11]. Based on these different challenges in cancer research, we strongly believe a combination of AgNPs being cytotoxic to cancerous cells and large anisotropic AuNPs with PTT features would provide a multifaceted treatment option. With many more applications being envisioned, new synthetic routes are continuously evolving in the field of metal nanoparticles as indicated by the number of publications reported in the last 20 years [12]. The route herein, however, is distinct in that no cytotoxic reducing agents or stabilizers are used–at all–in the synthesis of the bimetallic Ag-Au NPs while preserving or improving the properties of each metallic component vs analogous hybrid nanostructures known hitherto in the literature.

Surface plasmon resonance (SPR) measurements are often useful in distinguishing segregated from alloyed bimetallic NPs. A plasmon resonance band whose absorption maximum lies between those of Au and Ag NPs indicates the formation of alloyed nanoparticles, whereas two or more plasmonic absorption bands with varied aspect ratios indicate the formation of separate or segregated bimetallic NPs with tunable absorption [13,14]. Alloyed Au-Ag NPs display little segregation, mostly because they exhibit a similar lattice parameter of 4.09 Å and 4.08 Å, respectively [15]. With this understanding, NPs with similar lattice parameters and crystal structures follow a similar pattern of nucleation and growth [16,17]. However, when nucleation and growth are separated through seeding, epitaxial growth of the feed precursor over the seed can be controlled to retain the optical properties of each material. After showing similar work, Gu et al. proposed that bimetallic NPs are formed by catalytic sites on the Au seed surface, leading to a reduction of Ag^+^ ions, which then form the nucleation center for subsequent reduction of Ag^+^ ions [18]. More recently, a new approach was proposed by Goudelli and Pratsinis that explained theoretically the process of nanoparticle twinning, occurring through the coalescence of silver and gold NPs at different temperatures—resulting in segregated, binary NPs [19]. This report outlines a simple two-step photochemical strategy for generating a conjoined composite material comprised of Ag-Au or Au-Ag NPs, referred to herein as “nanotwin” particles with plasmonic absorption in the visible and near infrared (NIR) region. Ag-Au nanotwins are prepared using the environmentally-benign, biologically-derived polymer CS. Two key aspects of this method are the use of CS as an NP stabilizer and photoirradiation for photoinduced reduction of gold (I) and silver (I) molecular precursors or salts to form NPs. This procedure provides an avenue for making different sizes (~30–100 nm) of hybrid nanostructures suitable for different applications. During both the seed and feed processes, which result in the formation of hybrid nanoparticles, no harsh chemical reducing agents and/or stabilizers are utilized.

## 2. Materials and Methods

The synthetic procedure was begun by making a stock solution of 1.0 wt% medium molecular weight chitosan (CS, medium molecular weight, 85% deacetylated) prepared in deionized water using a 1 M acetic acid solution to adjust the pH to 4. The solution was stirred overnight prior to use to allow it to dissolve completely. The CS solution was then dialyzed overnight to remove excess acetic acid. Stock solutions of AgNO_3_ were made by dissolving AgNO_3_ in DI H_2_O to make 20 mL of 1 mM or 10 mM solution. Synthesis of the nanotwins was accomplished using a seeded-growth approach. Gold seed solution was prepared using chloro(dimethyl sulfide)gold(I) ((CH_3_)_2_SAuCl) (1 mM or 10 mM) dispersed in deionized water to make 20.0 mL of solution followed by vortexing to homogenize the mixture into a suspension of fine particles.

The photoirradiation was carried out by a 450-W medium-pressure mercury vapor lamp –Source 1 (HANOVIA Specialty Lighting LLC, Fairfield, NJ, USA)–whose total energy output is described by the manufacturer to be approximately 40–48% in the ultraviolet region and 40–43% in the visible region, with the remainder in the infrared region. Photoirradiation was used to facilitate the formation of seeds followed by the slow growth of the feed under similar conditions. Small seeds were achieved using a Spectrum 100 UVA mercury lamp fitted with a 15-W/cm^2^ power output and a fiber optic light guide equipped with an IR filter—Source 2 (DoctorUV.COM, Redondo Beach, CA, USA).

A Perkin–Elmer Lambda-900 double-beam UV/Vis/NIR absorption spectrophotometer (PerkinElmer, Shelton, CT, USA) was used for recording SPR peaks/bands of solutions containing Au, Ag, and hybrid Au-AgNPs. Electron microscopy was performed using an FEI Co. Tecnai G2 F20 S-Twin 200 keV field-emission Scanning Transmission Electron Microscope (S/TEM). A 1-nm STEM probe allows for an imaging resolution of 0.19 nm, and a high angle annular dark field detector (HAADF) allows for Z-contrast imaging in STEM mode at high resolution (FEI, Hillsboro, OR, USA).

### Synthesis Procedure

A typical procedure for making Au-Ag hybrid nanoparticles: The detailed procedure for making Au seeds is listed elsewhere [20]. In short, the procedure involves making 1.0 wt% of chitosan (CS, medium molecular weight, 85% deacetylated) solution first at pH 4.0. The solution was then stirred overnight prior to use. Chitosan solution was then dialyzed to remove excess acetic acid. Gold seed solution was prepared by dispersing and irradiating the solid chloro(dimethyl sulfide)gold(I) ((CH_3_)_2_SAuCl) (1 mM or 10 mM) in the required volume of CS solution. Upon irradiation of the required amount of Au(I) precursor in the CS solution, gold nanoseeds (AuNPs) were formed.

Gold nanoseeds were developed into Ag-Au nanotwins by addition of AgNO_3_ as a feed precursor followed by irradiation in an ice-cold bath. In the first step, isotropic (spherical) or anisotropic (non-spherical) AuNPs were generated by photochemical irradiation of an Au(I) precursor in a CS solution; in the second step, aqueous AgNO_3_ was added to facilitate slow growth of AgNPs on the surface of the AuNP seed through continuous irradiation and stirring in an ice bath.

Making small Au-Ag hybrid nanoparticles (small nanotwins): To make the Au seeds, 1.0 mL of the 1 mM (CH_3_)_2_SAuCl suspension followed by 1.0 mL of 1.0 wt/wt% CS solution was added to a borosilicate glass vial and irradiated using Source 1 for 60–90 min at 0–4 °C in an ice-water bath while continuously stirring to obtain isotropic NPs. The same isotropic material containing small seeds can be obtained by irradiating for 360 s using light Source 2 at room temperature. To obtain the nanotwins, one equivalent of the seed product was put in a different vial followed by different equivalent ratios each of the AgNO_3_ and CS solutions. The mixture was then placed in an ice-water bath in a photochemistry box (Source 1) and irradiated for another 5, 15, or 30 min with continuous stirring to obtain the nanotwins.

Making large Au-Ag hybrid nanoparticles (large nanotwins): The size and SPR of the nanotwins were found to be tuned by controlling irradiation times of both the seed and feed. The procedure for making the large nanotwins was exactly the same as that for the small nanotwins except for changing the irradiation during seed formation. The irradiation time was 120 min compared to 60–90 min for the small seed generation. The feed procedure followed for generating large nanotwins is also very similar, as described above, for the formation of small hybrid Au-AgNPs. The large anisotropic seeds resulted in larger size bimetallic NPs. The plasmonic absorption was also clearly distinguishable between small and large size bimetallic NPs.

## 3. Results and Discussion

**Figure 1:**
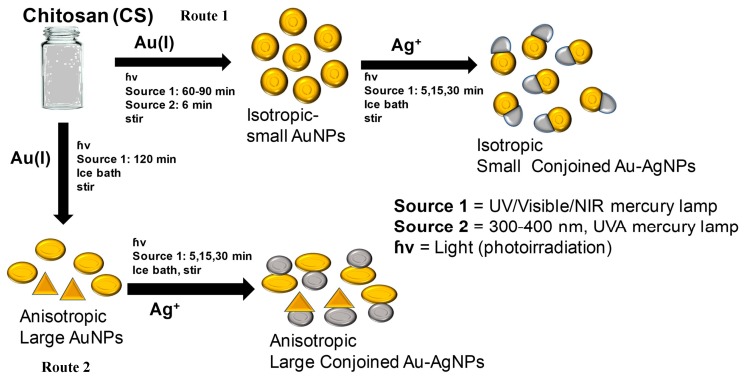
Schematic representation of the synthesis of hybrid Au-Ag nanoparticles of different sizes via a photochemical process by two different routes (1 and 2). The first step in both routes is the formation of AuNPs (seed), whereas the second step is the formation of conjoined nanotwins, hybrid Au-AgNPs, in the presence of an Ag^+^ feed.

The full schematic representation for the formation of both large and small hybrid bimetallic conjoined NPs is shown in Figure 1. The scheme depicts the formation of conjoined Au and Ag NPs with an average size of ~30 nm for small nanotwins and ~70 nm for large nanotwins. Irradiation time was found to correspond with AgNP size. We propose a photoinduced reduction of both gold and silver monovalent precursors during the formation of hybrid nanoparticles, similar to the mechanism proposed by Alarcon et al. [21] and El Sayed [21] for the photochemical formation of gold and silver nanoparticles, respectively. In Au^+^ photoreduction, the Au precursor, a labile Au^+^ complex, easily undergoes photoinduced reduction to form Au^0^. For AgNPs, the silver(I) salt undergoes a similar photoinduced reduction from Ag^+^ to Ag(0). Both 1-e^−^ photochemical reductions occur in the absence of any dedicated reducing agents typically used in the literature (e.g., NaBH_4_) so only radical formation from the water solvent and/or CS stabilizer generated via the UV source must be responsible for the photoelectron needed for the photoreduction of M(I) to M(0).

**Figure 2:**
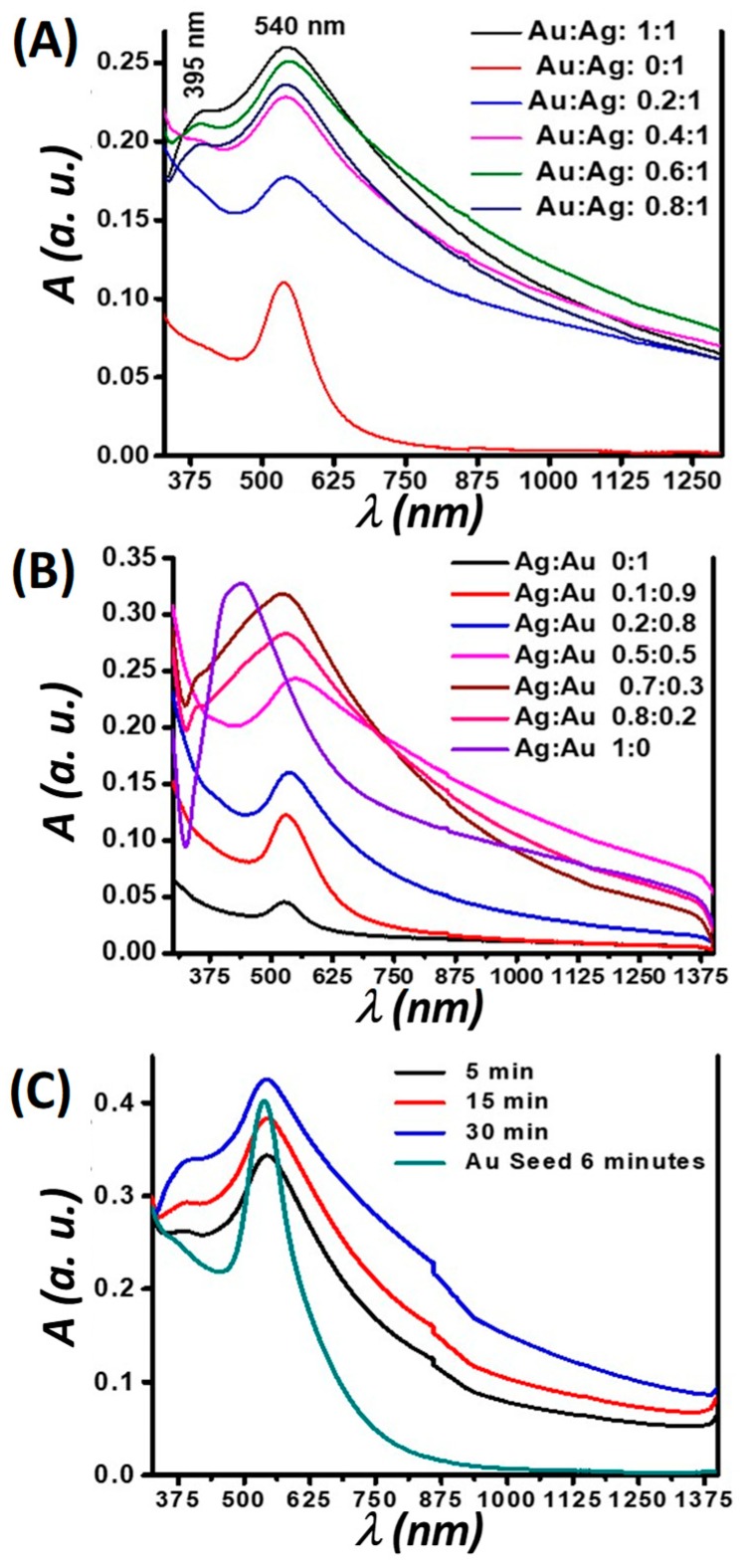
Plots of absorption spectra of the Au-Ag nanotwin NPs prepared using 1 mM of the Au(I) precursor as the seed, irradiation using Source 1 for 90 min in (**A**,**B**). The volume of 10 mM AgNO_3_ feed solution added is varied for constant volumes of 1 mM Au in (A) and relative variation of seed to feed in (**B**). (**C**) Shows SPR spectra of 1 mM Au(I) precursor seed created by 6 min of irradiation using Source 2 followed by time-dependent irradiation of the same seed solution at a 1:1 volume ratio with 10 mM AgNO_3_ feed using Source 1. All ratios represented here in A and B are volume ratios of Au(I) and Ag(I) precursor concentrations.

Representative SPR data that demonstrate the formation of small nanotwins are shown in Figure 2. Absorption measurements of the nanotwins showed spatially-distinct absorption bands centered at 400 nm and 545 nm (Figure 2). In Figure 2A, 1 mM of the Au(I) precursor seed irradiated for 90 min formed small AuNPs with an absorption band centered near 550 nm. Increasing the amount of 10 mM Ag(I) precursor relative to a constant AuNP seed concentration leads to the formation of AgNPs, whose absorption band was centered at 400 nm, along with a broadening and intensifying of the AuNP absorption band. It is noteworthy that no peak shifts were seen in any cases, unlike what is observed in most photochemical processes where isotropy is broken as the reaction progresses and a secondary absorption band forms that red-shifts as the nanoparticle growth progresses. Based on literature and data analysis, we assume that–due to competing processes–growth on a single site of the nanoparticles is highly preferred to that over multiple sites and in different directions. This growth could also be auto-catalytic at the initial Ag nucleation site and is preferred over other sites on the Au seed. Many literature precedents have demonstrated nanorod formation in a similar fashion by forming seeds and subsequent growth in one direction [22].

In Figure 2B, similar results when decreasing the amount of the seed (Au) relative to an increasing amount of the feed (Ag) are demonstrated. It is evident from these results that–when the AuNP seed was doped with trace amounts of Ag^+^ feed–there was a dramatic increase in the peak attributed to the AuNP; as the relative amounts of Ag increased, another peak emerged (Figure 2B, (Ag: Au = 0.7:0.3)). The sharp increase may be due to Ag^+^ ions acting in part as growth directors of residual unreacted Au in the system [23]. Figure 2C shows time-based data for the formation of nanotwins by first preparing the seed using a fiber optic lamp (Source 2) to obtain monodispersed small AuNPs, comprising a narrow full width at half max (FWHM) at 541 nm. Irradiation was done for 360 s followed by the slow growth of feed by using Source 1 in an ice bath with the irradiation time used as a growth control. As seen in Figure 2C, the AgNP peak begins to emerge (at about 400 nm) within 5 min of irradiation. Unlike in the other situations in Figure 2A,B, there is no sharp rise in the Au absorption band taking place in Figure 2C, indicating more monodispersity evident from this peak. In all cases, there was a general broadening of the peaks attributed to the nanotwins, which suggests the disturbance of isotropy as nanotwin formation occurrs, leading to larger-size NPs.

It is evident that the SPR bands for both Au and AgNPs exhibit no notable shift over time, which is a significant indicator of the NPs retaining their characteristic optical properties. Stronger absorption by AuNPs relative to AgNPs is hypothesized herein to be due to the fact that AuNPs have a stronger extinction coefficient than AgNPs [24,25,26].

**Figure 3:**
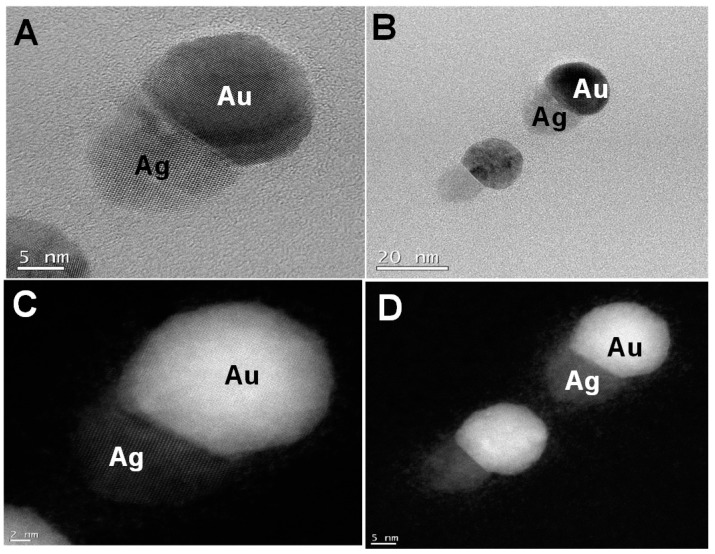
Bright field (**A**,**B**) and dark field (**C**,**D**) TEM images of the conjoined Au-Ag nanotwins formed by irradiation of the seed for 90 min in the seeding step and 30 min in the second step.

**Figure 4:**
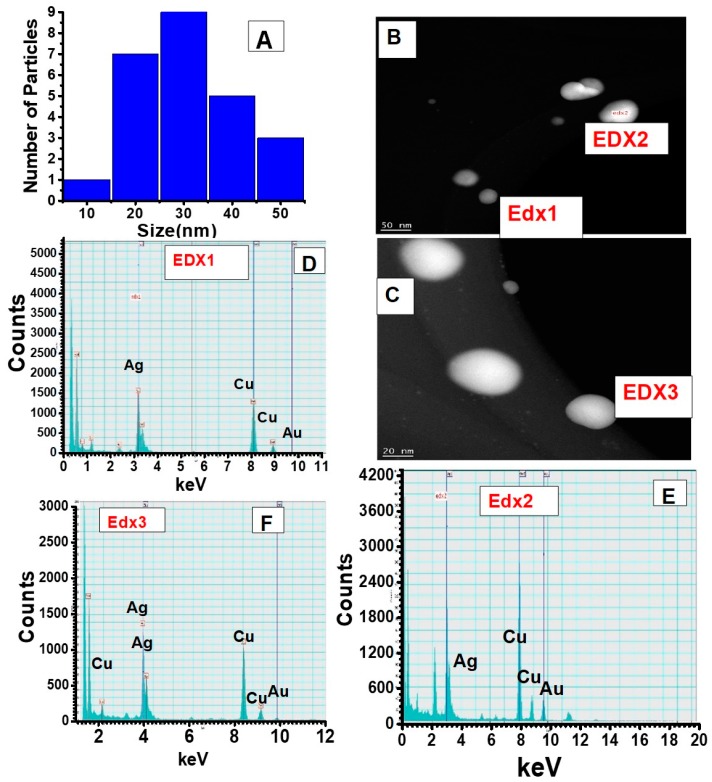
Elemental characterization and size distribution of conjoined Au-Ag nanotwins formed by irradiation of the seed for 90 min in the seeding step and 30 min in the second step. (**A**) Size distribution was obtained from the TEM images of Figure 3. (**B**–**F**) Energy dispersive X-ray spectroscopy data obtained from different spots of the bimetallic NPs.

The formation of small nanotwins as described in the scheme (Figure 1) following Route 1 is experimentally demonstrated from Figure 3. For comparison, Figure 3A,C show the bright field and the dark field images of the small nanotwins, respectively. The AuNPs appear brighter in the dark field than Ag. A similar observation is seen for Figure 3B,D, indicating that these are two distinct materials. From the bright field TEM images, the average size of the NPs has been determined to be about 30 nm, as shown in Figure 3A. For each NP, the average calculated size of Au (assuming each metal NP is completely spherical) is about 60–68% of the overall size of the nanotwin, forming a dumbbell-shaped NP. It may also be due to the relatively larger size of the AuNPs compared to AgNPs in each nanotwin (Figure 4A–D). Figure 4 shows the particle distribution profile obtained from the TEM images from Figure 3, which clearly shows an average size of 30 nm for these bimetallic nanotwin particles. The elemental characterization for understanding the Au/Ag distribution was performed from EDS spectra and spot images of the small nanotwins (Figure 4). The EDS data in Figure 4 very clearly show the clear presence of both Ag and AuNPs in each nanotwin, confirming the formation of a bimetallic nanotwin as represented in Figure 1. Based on the EDS data in Figure 4, we deduce that the particles under analysis exhibit a much higher concentration of silver compared to gold. At this stage, there is no empirical evidence to support the data other than a higher initial concentration of Ag (10 mM) compared to the 1 mM Au concentration. We are currently working on fully understanding these results. However, irradiating the feed for more than one hour did not yield a higher concentration of nanotwins as expected, but rather a mixture of a highly-polydispersed and aggregated material was formed, composed of larger content of Ag and smaller content of Au, as indicated from the data shown in Figure S1 (refer to the Supplementary Materials). Based on the TEM and EDS data, we assume that the formation of a core-shell structure based on the findings in the literature —where it was noticed that the core constituted less than 5% of the total count while the shell was seen to constitute more than 95% on EDS spectra upon prolonged formation of the shell [18].

**Figure 5:**
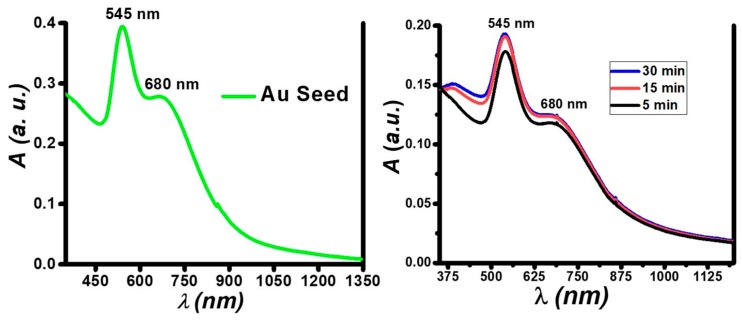
UV-Vis spectra of large, anisotropic AuNPs (left) and time-based formation of Au-Ag nanotwins (right) using the large, anisotropic AuNPs as seeds. The left AuNP seeds are generated by irradiation of 1 mM Au(I) precursor for 120 min using Source 1. The right panel Au-Ag nanotwins are formed by irradiating a 1:1 solution containing the AuNP seed and 10 mM AgNO_3_using Source 1.

**Figure 6:**
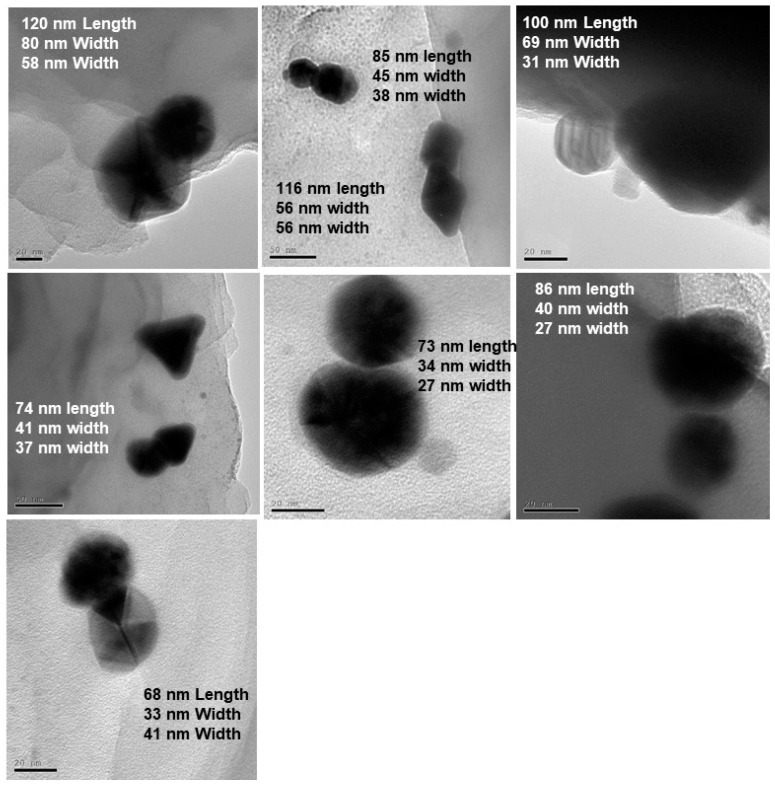
Bright field TEM images showing large Au-Ag nanotwins formed by 120 min of irradiation of large, anisotropic AuNPs seeds followed with 30 min of irradiation with Ag^+^ feed.

**Figure 7:**
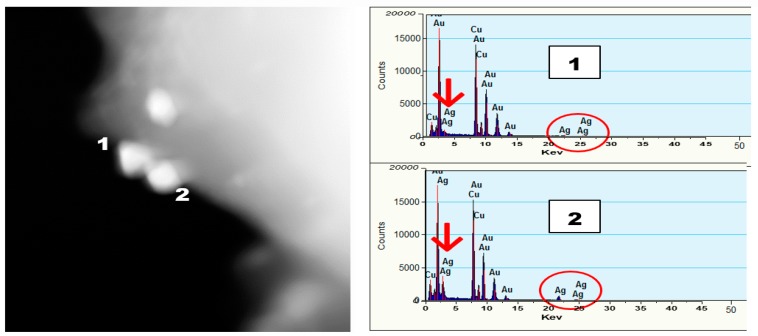
STEM images and EDS spectra for large bimetallic Au-Ag nanotwins. Left: Spot analysis images. Right: EDS spectra.

Figure 5 shows UV-Vis data for the formation of anisotropic AuNPs (left) and subsequent formation of Au-Ag bimetallic nanotwins in the presence of 10 mM AgNO_3_ upon additional 5, 15, and 30 min of irradiation using light Source 1 in an ice bath. The emergence of a peak at 393 nm is attributable to the formation of small AgNPs, and herein, we refer to it as a “nanobaby” on the anisotropic AuNPs surface. The increase in the peak intensity with irradiation time indicated the formation of more Ag “nanobabies” over the seed. Our data suggest that the concentration of “nanobabies” can also be tuned by varying the experimental parameters. Figure 6 shows TEM images obtained for the larger bimetallic Au-AgNPs as represented by Route 2 of the schematic representation (Figure 1). The STEM images and EDS data from Figure 7 rather clearly illustrate the formation of larger, anisotropic, bimetallic, and conjoined nanotwin Au-AgNPs. Multiple images collected for the 30-min irradiated feed sample give rise to particles larger than 70 nm in terms of average size of the bimetallic NPs. The anisotropic morphology suspected based on the SPR absorption (Figure 5) of the conjoined Au-AgNPs is clearly evident from and validated by these TEM images in Figure 6. Followed by TEM analysis, STEM images for analyzing the elemental composition were collected, as shown in Figure 7. The spot analysis from STEM images and the EDS data confirm the formation of conjoined, bimetallic, larger nanotwins with sizes larger than 50 nm. When the electron beam was focused on the triangular particle labeled “1” (Figure 7), little or no Ag was detectable near 3 keV (marked with the red arrow) and 22.5 keV (circled in red), and strong Au peaks were seen at 9, 11m and 13 keV, respectively. Upon focusing the beam on the particle labeled “2”, peak intensities for Ag increased significantly at 3 keV and 22.5 keV, indicating that particle”2” is silver while its counterpart “1” is Au. The strong Cu peaks noticed in the EDS spectra were attributed to the TEM grid.

Based on the multiple TEM images from Figure 6, Table 1 shows the correlation between the irradiation time and size of the nanotwin. The longest distance running across both nanoparticles has been considered as the length of the nanoparticle. In this case, the average size of the nanotwin is ~76 nm after 15 min of irradiation and ~100 nm after 30 min of irradiation with silver feed. The size variation as noticed in Table 1 indicates the tunability of the size of these conjoined nanotwin Au-AgNPs by varying the irradiation time.

## 4. Conclusions

In conclusion, this report demonstrates a simple and efficient photochemical strategy to form different sizes of Ag-Au hybrid conjoined nanoparticles (herein, we refer to them as “nanotwins”). The synthesis has been accomplished by using two different light sources and by varying the precursor concentration, reaction conditions, and irradiation times. This methodology resulted in the formation of hybrid-segregated nanotwins with individual optical properties unique to each metal. We strongly believe that the bifunctional conjoined NPs prepared by applying this strategy could find applications in catalysis, surface-enhanced Raman spectroscopy (SERS) based imaging, and in biomolecule sensing. With the ability to tune the SPR of these hybrid nanoparticles, we believe these bimetallic NPs with SPR across the visible and NIR regions would find applications in photothermal therapy -- with considerable advantages compared to individual monometallic gold or silver nanoparticles—given the favorable SPR of the former and cytotoxicity to cancer cells at suitable doses of the latter. We also presume that Ag-Au nanotwins are promising adjuvants, which could be used alongside photothermal therapy to treat multi-drug-resistant (MDR) cancers.

## Figures and Tables

**Table 1:** Diameter of large Au-Ag nanotwins determined from TEM images after 15 min and 30 min of irradiation. For non-spherical particles, the longest dimension refers to the length of the particle considered for the size measurements herein.

Irradiation Time (min)	Material	Size of the Nanoparticles (nm)									Average Size (nm)
15	Ag	27	56	37	38	30	25	23	21	27	31.3
	Au	34	56	41	45	35	30	30	33	40	38.2
	Au-Ag	73	116	74	65	85	80	48	58	86	76.1
30	Ag	41	31	22	53	58					41
	Au	33	69	46	73	80					60.2
	Au-Ag	68	100	105	113	120					101.2

## Supplementary Materials

The following are available online at https://www.mdpi.com/2079-4991/9/4/596/s1: Figure S1: Elemental characterization of conjoined Au-Ag nanotwins formed by prolonged irradiation (more than one hour) of the seed in the seeding step and 30 min in the second (feed) step.
