# Supplementary material for: Facile Photochemical Syntheses of Conjoined Nanotwin Gold-Silver Particles within a Biologically-Benign Chitosan Polymer
Source: Nanomaterials (Basel). 2019 Apr 11;9(4):596. doi: 10.3390/nano9040596 (PMC6523650; doi:10.3390/nano9040596)
Supplement: Supplementary file 1 [file nanomaterials-09-00596-s001.pdf]

Supplementary Materials

Facile Photochemical Syntheses of Conjoined Gold-Silver Nanoparticle within a Biologically Benign Chitosan Polymer

Daniel K. Korir [1], Bharat Gwalani [2], Abel Joseph [1], Brian Kamras [1], Ravi K. Arvapally [1], Mohammad A. Omary [1,*] and Sreekar B. Marpu [1,*]

[1] Department of Chemistry, University of North Texas, Denton, TX 76203, USA; DanielKorir@my.unt.edu (D.K.); AbelJoseph2@my.unt.edu (A.J.); BrianKamras@my.unt.edu (B.K.); RaviArvapally@my.unt.edu (R.K.A.)

[2] Department of Materials Science and Engineering, University of North Texas, Denton, TX 76203, USA; BharatGwalani@my.unt.edu

* Correspondence: Mohammad.Omary@unt.edu (M.A.O.); sreekarbabu.marpu@unt.edu (S.B.M.)

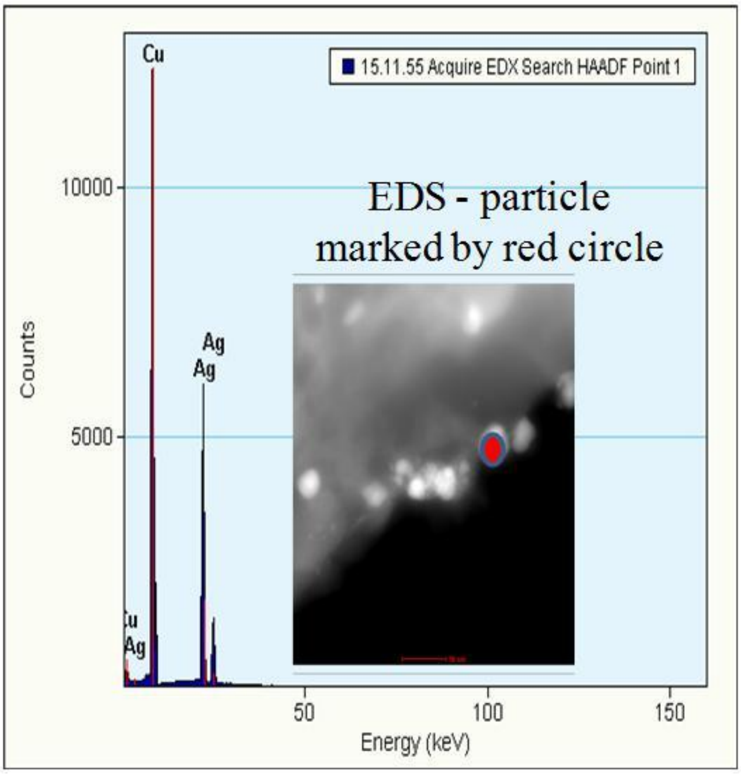

Figure S1. Elemental characterization of conjoined Au-Ag nano-twins formed by prologoned irradiation (more than one hour) of the seed in the seeding step and 30 min in the second step.
